# Hepatitis B in Women: Domestically and Internationally^1^

**DOI:** 10.3201/eid1011.040624_02

**Published:** 2004-11

**Authors:** Cindy Weinbaum, Susan Goldstein, Julie Subiadur

**Affiliations:** * Centers for Disease Control and Prevention, Atlanta, Georgia, USA;; † Denver Public Health Department, Denver, Colorado, USA

**Keywords:** hepatitis B, epidemiology, vaccination programs, prevention and control, sexually transmitted diseases

Globally, hepatitis B virus (HBV) infection is a major cause of infectious disease–related death, causing approximately 620,000 deaths annually. Without hepatitis B vaccination, an estimated 1.4 million HBV-related deaths would occur in the 2000 birth cohort over the lifetime of the cohort. HBV infections acquired in the perinatal and early childhood periods account for 21% and 48%, respectively, of HBV-related deaths worldwide. Thus, routine vaccination of infants and children serves as the basis for a global hepatitis B prevention program.

In 1992, the World Health Organization recommended that hepatitis B vaccine be included in childhood immunization programs in all countries, but because of financial constraints, many countries were unable to initially implement this recommendation. In 1999, a global initiative began to make hepatitis B vaccine available to children living in 69 of the world's poorest countries, and by the end of 2003, routine childhood hepatitis B vaccination was included in national immunization programs in >151 countries. However, many countries, mainly in sub-Saharan Africa, have not yet introduced the vaccine, and coverage with the three-dose vaccination series remains low in many countries that have introduced the vaccine. When all countries have introduced the vaccine and coverage with the three-dose vaccination series reaches 90%, up to 84% of global HBV-related deaths will be prevented.

## Hepatitis B in the United States

In the United States, an estimated 5% of the civilian, noninstitutionalized population has serologic evidence of past or present HBV infection, and 0.4%–0.5% have chronic infection and are the primary source of infection for others. From 1990 through 2002, the incidence of reported acute hepatitis B declined 67%. The incidence of acute hepatitis B among men has been consistently higher than among women. In 1990, the incidence among men and women was 9.8 and 6.3 per 100,000, respectively; in 2002, the incidence was 3.7 and 2.2 per 100,000, respectively. Overall, incidence among women has declined more than among men. Trends in acute hepatitis B reflect poor vaccination coverage among persons who engage in high-risk behavior.

Persons at high risk for HBV infection often seek health care in settings in which vaccination services could be provided. During 1996–1998, approximately half of persons with reported acute hepatitis B previously had been treated for a sexually transmitted disease (STD) or incarcerated: 89% of injection drug users, 35% of men who have sex with men, and 70% of persons with multiple sex partners with reported acute hepatitis B had been previously incarcerated or treated for an STD. Both STD clinics and correctional facilities are settings in which hepatitis B vaccination services are recommended.

## Programmatic Success in High Risk Settings

In August 1999, Denver Public Health (DPH) began offering hepatitis B vaccine to adults at high risk in the public STD clinic. Initial funding for the vaccine was first allocated by the Denver City Council. Patients were asked if they had a history of hepatitis B vaccination or disease and questioned about risk behavior; no serologic screening was done. The selective vaccination process was cumbersome, and clinicians required frequent reminders to implement it. Of clients seen in the STD clinic, 58% accepted the vaccine and were directed to receive it in the immunization clinic in the same building. Of clients who agreed to the free vaccine, 29% left before receiving it. Procedures changed when additional funding was secured in January 2002. Client selection was discontinued, and all clients of the STD and HIV Counseling and Testing clinics were offered vaccine, which increased its initial acceptance to 77%. Vaccination rates were further improved by having personnel available to vaccinate clients on site, before they left the clinic.

DPH used a vaccine registry, adapted from one implemented to track pediatric vaccinations, to assess clients' vaccination status before doses were given. The results indicated that clients were not differentiating between vaccinations and various other tests or medications in self-reporting of immunization status. Use of the vaccine registry was crucial for evaluating completion rates and eliminating revaccination of persons already immunized.

A highly successful hepatitis B vaccination program can be established within another public health infrastructure. The process requires commitment from all involved programs because changes in service delivery are needed to accommodate vaccination. The largest issue confronting programs is continued funding for vaccine.

